# Improved opioid prescribing in primary care: protocol for a cluster randomised pragmatic trial

**DOI:** 10.1136/bmjopen-2025-110818

**Published:** 2025-12-17

**Authors:** Cecilia Krüger, Johan Franck, Jonas Hällgren, Sandra af Winklerfelt Hammarberg, Christer Norman, Åsa Niper, Jeanette Westman

**Affiliations:** 1 Department of Neurobiology, Care Sciences and Society, Karolinska Institutet, Huddinge, Sweden; 2 Department of Clinical Neuroscience (CPF), Karolinska Institutet, Stockholm, Sweden; 3 The Stockholm Centre for Dependency Disorders, Region Stockholm, Stockholm County, Sweden; 4 Academic Primary Care Centre, Region Stockholm, Stockholm County, Sweden; 5 Department of Health Care Sciences, Marie Cederschiöld Högskola, Stockholm, Sweden

**Keywords:** Primary Care, Primary Health Care, Guideline Adherence, Prescriptions, Randomized Controlled Trial

## Abstract

**Introduction:**

Opioid analgesic medications play a critical role in pain management but are associated with significant risks, including addiction. General practitioners in primary care account for a substantial proportion of opioid prescriptions, and prescribing practices may not always fully align with clinical guidelines. Given the limited evidence supporting long-term opioid use for chronic non-cancer pain, there is a pressing need for interventions that promote safer, guideline-concordant prescribing. The *Smarta Val* (Smart Choices) trial will evaluate whether a new multicomponent intervention, comprising an educational seminar, written materials and feedback on prescribing over 12 months, can improve opioid prescribing practices in primary care.

**Methods and analysis:**

This cluster randomised pragmatic trial will assess changes in opioid prescribing across primary healthcare centres (PHCCs) in Stockholm, Sweden. Consenting PHCCs will be randomised 1:1 to either the intervention group, receiving the multicomponent intervention, or the active control group, receiving a leaflet on prescribing recommendations. A sample size of 24 PHCCs per group is required to detect differences in opioid prescribing between groups. A third group of non-randomised observational reference PHCCs will be included to provide contextual information on prescribing practices during the study period. Data sources include regional healthcare databases, baseline and 12-month follow-up questionnaires, and an intervention delivery form. The primary outcome is the change in prescription of opioids at 12 months. Secondary outcomes are the change in prescription of opioids at 24 months and the change in the specific opioid substances prescribed at 12 months.

**Ethics and dissemination:**

The study has been approved by the Swedish Ethical Review Authority (Dnr 2021-06739-01). Participation in the study requires informed consent from PHCC managers in the intervention and active control groups. Results will be disseminated through international peer-reviewed journals and conference presentations.

**Trial registration number:**

NCT05577026.

**STRENGTHS AND LIMITATIONS OF THIS STUDY**

- This cluster randomised trial evaluates a brief, scalable intervention comprising an educational seminar, written materials and feedback on opioid prescribing over 12 months.
- The intervention is delivered at the level of the primary healthcare centre (PHCC), targeting prescribing practices among primary healthcare professionals and reinforcing adherence to clinical guidelines.
- A two-year preintervention period and a 36-month postintervention follow-up allow for robust analysis of long-term prescribing trends.
- Inclusion of three PHCC groups (intervention, active control and observational reference) enhances internal validity and enables differentiation between intervention effects and secular trends.
- The trial is conducted among PHCCs in Stockholm that opted into participation, which may limit generalisability; however, the inclusion of an observational reference group supports broader contextual interpretation.

## Introduction

 Opioid analgesic medications are widely used for pain management, although caution is recommended when prescribing opioids due to the risk of adverse health outcomes, including addiction and opioid-related deaths.^1^,^3^ General practitioners (GPs) in Swedish primary healthcare account for an estimated one-third of opioid prescriptions,^4^ and although opioid prescription is declining,^5 6^ there is still room for improvement in adherence to prescription guidelines.^3 4 7^

International and Swedish clinical guidelines recommend prescribing opioids at the lowest effective dose for the shortest possible duration, as risk increases with prolonged use.^18^,^10^ Additionally, there is insufficient evidence supporting the long-term effectiveness of opioid treatment for chronic (ie, lasting for more than three months) non-cancer pain.^10^,^12^ Therefore, opioids should be reserved for selected cases within a multimodal treatment approach.^8^ Despite these clear recommendations, inconsistent application in practice persists.^7^

Prior research has identified several strategies that can effectively influence prescribing behaviour. These include social norm feedback or ‘nudging’,^13^ audit and feedback mechanisms,^14^ and feedback that is delivered frequently, by a figure of authority, and linked to clearly defined goals and action plans.^15^ Strategies to enhance prescribers’ knowledge through dissemination, education and training may improve guideline adherence.^7^ Interventions specifically targeting opioid prescribing have shown mixed results; however, those demonstrating positive outcomes often incorporate educational components (eg, academic detailing with evidence from trusted sources, workshops, continuing medical education),^16^,^19^ written materials (eg, policy documents, pamphlets),^20^,^22^ and/or audit and feedback tools (eg, memos, email reminders).^21 23 24^ These three components were therefore integrated into the intervention in the *Smarta Val* (Smart Choices) trial.

Despite promising findings in some studies, the overall quality of evidence supporting educational, policy and regulatory interventions aimed at modifying prescribing behaviour remains low.^25^ In particular, there is a lack of robust research on prescriber education targeting healthcare professionals who manage patients with chronic non-cancer pain.^26^ Given that primary care plays a central role in the initial and ongoing management of chronic pain, this represents a knowledge gap in the literature.

Furthermore, existing opioid-related interventions have been limited in both scope and context. Most originate from the US, and few have been implemented in primary healthcare settings. To date, no studies have been identified in Scandinavian countries, where differences in healthcare systems, such as regulatory frameworks, professional roles, clinical guidelines and reimbursement mechanisms, may significantly influence both implementation and effectiveness. Broader systemic and societal factors, such as healthcare access and equity, further limit the generalisability of US-based findings to the Swedish context.

To address this evidence–practice gap, we developed an intervention for Swedish primary care that integrates components with demonstrated potential to influence prescribing behaviour in real-world settings and be incorporated into routine clinical practice, aligning with the pragmatic nature of the trial. We chose to include both active control and observational reference primary healthcare centres (PHCCs) to enable comparison with standard care while also capturing real-world prescribing patterns, enhancing both internal and external validity.

### Objective

The overall objective is to evaluate, in a cluster randomised trial, the effectiveness of a new intervention that includes an educational seminar, written materials and 12 months of bimonthly prescribing feedback on opioid prescribing in primary healthcare. The intervention group will be compared with an active control group, receiving a leaflet on prescribing recommendations, and an observational reference group, continuing with usual care practices.

## Methods and analysis

### Design

This pragmatic cluster randomised trial focuses on evaluating the real-world effects of the intervention in the primary healthcare setting.^27^ Managers of invited PHCCs will consent for their centre to be randomised in the trial, which targets opioid prescribing behaviours among healthcare professionals. Prescription of opioids to registered adult patients at each PHCC, regardless of diagnosis or indication, will be followed in regional healthcare databases before and after the intervention period. The trial also includes an examination of intervention delivery, which will be reported separately. The Standard Protocol Items: Recommendations for Interventional Trials checklist^28^ was used to guide the reporting of this protocol.

### Setting

The study will be conducted among PHCCs in Stockholm County that hold a primary care contract with Region Stockholm, which is the governing body responsible for healthcare in the county.

Primary healthcare in Sweden is a comprehensive, regionally organised system comprised of public and private PHCCs within the tax-financed universal healthcare system.^29^ It is often the first point of contact for patients,^29^ and provides general medical consultations, preventive medicine and chronic disease management.^30^ In Sweden, patients are formally registered with a specific PHCC, a system that supports both continuity of care and capitation-based reimbursement.

### Eligibility and recruitment

The following steps will be used to identify eligible PHCCs:

- Identify PHCCs in Stockholm County with more than 3000 registered adult patients (≥18 years) as of December 2022 (n=207).
- Among these, select PHCCs in the top 50% of opioid prescription volume, measured using defined daily dose (DDD) per registered patient for all opioids classified under Anatomical Therapeutic Chemical (ATC) code N02A. For these high-prescribing PHCCs (n=105), the prescribing range corresponds to 0.137–0.491 DDDs per registered patient.
- Send study invitations via email to managers of PHCCs meeting the above criteria.

Research coordinators will confirm eligibility with interested PHCC managers through verbal or written contact prior to obtaining written informed consent (online supplemental figure 1). Detailed eligibility criteria are presented in table 1.

**Table 1:** Eligibility criteria for the *Smarta Val* trial

Category	Criteria
Inclusion	Holds a primary care contract with Region Stockholm and is thus connected to regional healthcare databases (VAL) Employs at least two full-time general practitioners
Exclusion	Has been in operation for less than 12 months

Recruitment strategies include contacting PHCC managers through up to three follow-up emails and three phone calls.

### Participant groups and interventions

The *Smarta Val* trial includes three groups of PHCCs. See table 2 for a full description of the study groups, intervention components and targeted behaviour change strategies.

**Table 2:** Description of trial participant groups, intervention components and proposed behaviour change strategies

Component	Description of component	Behaviour change strategies
**Intervention group**		
90-minute educational seminar	∼60-minute PowerPoint presentation of published peer-reviewed evidence and recommendations on pain management and opioid prescription. ∼7-minute video recorded by a key opinion leader on pain treatment.	Academic detailing Inclusion of experts in the field Presentation by a figure of authority Group discussion
	∼10-minute presentation and discussion of opioid prescribing at the PHCC level and comparison of prescription levels to nearby PHCCs.	Audit of prescription trends Benchmarking to nearby PHCCs Leveraging of social norms
	∼10-minute discussion of prescribing routines at the PHCC with recommendations for how to improve practices at the clinic.	Barrier identification Intention formation (common routine) Team building Encouragement
Written materials	Physical and electronic copies of written materials, including: Example patient record templates for prescribing and deprescribing opioids. Half-page reminder summarising Wise List^36^ opioid recommendations. Longer and simplified patient–provider agreements.	Visual reminder with references Tools to be used by prescribers and for potential use with patients
Prescription feedback	Bimonthly emails over 12 months, including graphs of opioid prescription (overall DDD and substance-specific) at the PHCC.	Reminders Self-monitoring Leveraging of social norms
**Active control group**		
Leaflet on prescribing recommendations	Single-page leaflet summarising Wise List^36^ opioid recommendations and links to national^10^ and regional^35^ guidelines, emailed to PHCC managers.	Visual reminder with references
**Observational reference group**		
Usual care	Continue with usual care practices; no interventions administered.	NA

DDD, defined daily dose; NA, not applicable; PHCC, primary healthcare centre.;

### Intervention group

The intervention consists of a 90-minute educational seminar, written materials and feedback on opioid prescribing at the PHCC. While the intervention primarily targets opioid prescribers, particularly GPs, all healthcare professionals with patient contact (eg, nurses, psychologists, receptionists and medical secretaries) at the PHCC are encouraged to attend the seminar. The intervention was developed by a multidisciplinary team encompassing expertise in general medicine and pain treatment, public health, pharmacy, psychiatry, psychology, physical therapy and communication. Data sources include:

- International recommendations on pain management and opioid treatment, including from Europe,^8^ the US Centers for Disease Control and Prevention,^1^ and the National Institute for Health and Care Excellence of the UK.^9^
- National recommendations by the Swedish National Board of Health and Welfare^12^ and Swedish Medical Products Agency.^10^
- Published peer-reviewed evidence by the Swedish Agency for Health Technology Assessment and Assessment of Social Services,^31^ and relevant review articles.^1132^,^34^
- County-specific resources, including Viss (Care Programmes and Information System in Stockholm),^35^ which provides clinical guidelines for primary care in Region Stockholm, and the Stockholm Drug and Therapeutic Committee’s Wise List,^36^ a formulary of recommended essential medicines.

Two GPs with experience in academic detailing are employed to deliver the seminar, which includes peer-reviewed evidence, a video recorded by a key opinion leader on pain treatment, general advice on tapering treatment and referral to addiction care, and presentation of prescribing patterns at the PHCC benchmarked against nearby PHCCs. Questions and discussion are encouraged throughout.

Written materials for the intervention group include provider-focused resources (patient record templates and a summary of the Wise List^36^ opioid prescribing recommendations), and patient–provider agreements for prescribers, which may also be used with patients. Physical copies will be distributed during the seminar, and all written materials and the presentation will be shared electronically afterwards. Bimonthly feedback on opioid prescribing, including overall and substance-specific DDDs, will be emailed to seminar attendees and PHCC managers for 12 months (online supplemental figure 2). The mailing list will be updated on request by the PHCC manager.

Consistent with the pragmatic trial design, participating healthcare professionals will determine the manner and frequency of study material use. During the seminar, PHCC managers will be asked to complete a baseline questionnaire. 12 months after the seminar, managers will be notified that the intervention period has ended and invited to complete a follow-up questionnaire.

### Active control group

Managers of PHCCs in the active control group will receive a single-page leaflet summarising the Wise List^36^ opioid prescribing recommendations and links to national and regional guidelines (Swedish Medical Products Agency^10^ and Viss^35^). The leaflet will be emailed once, without instructions for use. Managers will be contacted again after 12 months and will be asked to complete a follow-up questionnaire.

### Observational reference group

PHCCs in the observational reference group are not randomised, receive no study intervention and continue with usual care practices.

### Outcomes

#### Primary outcome

- Change in prescription of opioids at 12 months, measured by average DDD of opioids per patient month.

#### Secondary outcomes

- Change in prescription of opioids at 24 months, measured by average DDD of opioids per patient month.
- Change in the opioid substances prescribed at 12 months, measured by average DDD of opioids per patient month, based on ATC classification codes.

#### Other outcome measures

- Change in number of patients with opioid prescriptions at 12 months, measured as the number of patients with either new or continued opioid prescriptions.
- Change in number of patients with an initial (new) opioid prescription at 12 months, measured as the number of opioid-naïve patients initiating opioid therapy.

### Harms

There are standard routines to follow-up patient safety at each PHCC. Participating PHCCs have the possibility to report potential harms related to the trial to the study team. Risks to healthcare professionals and patients associated with the trial are anticipated to be minimal, given the educational nature of the intervention and its delivery at the PHCC level. While the study promotes guideline-concordant opioid prescribing, it does not advocate indiscriminate reductions, recognising the essential role of opioids in pain management. To mitigate the theoretical risk of overly restrictive prescribing or abrupt discontinuation, the intervention emphasises adherence to clinical guidelines and safe deprescribing practices.

### Participant timeline

**Figure 1:**
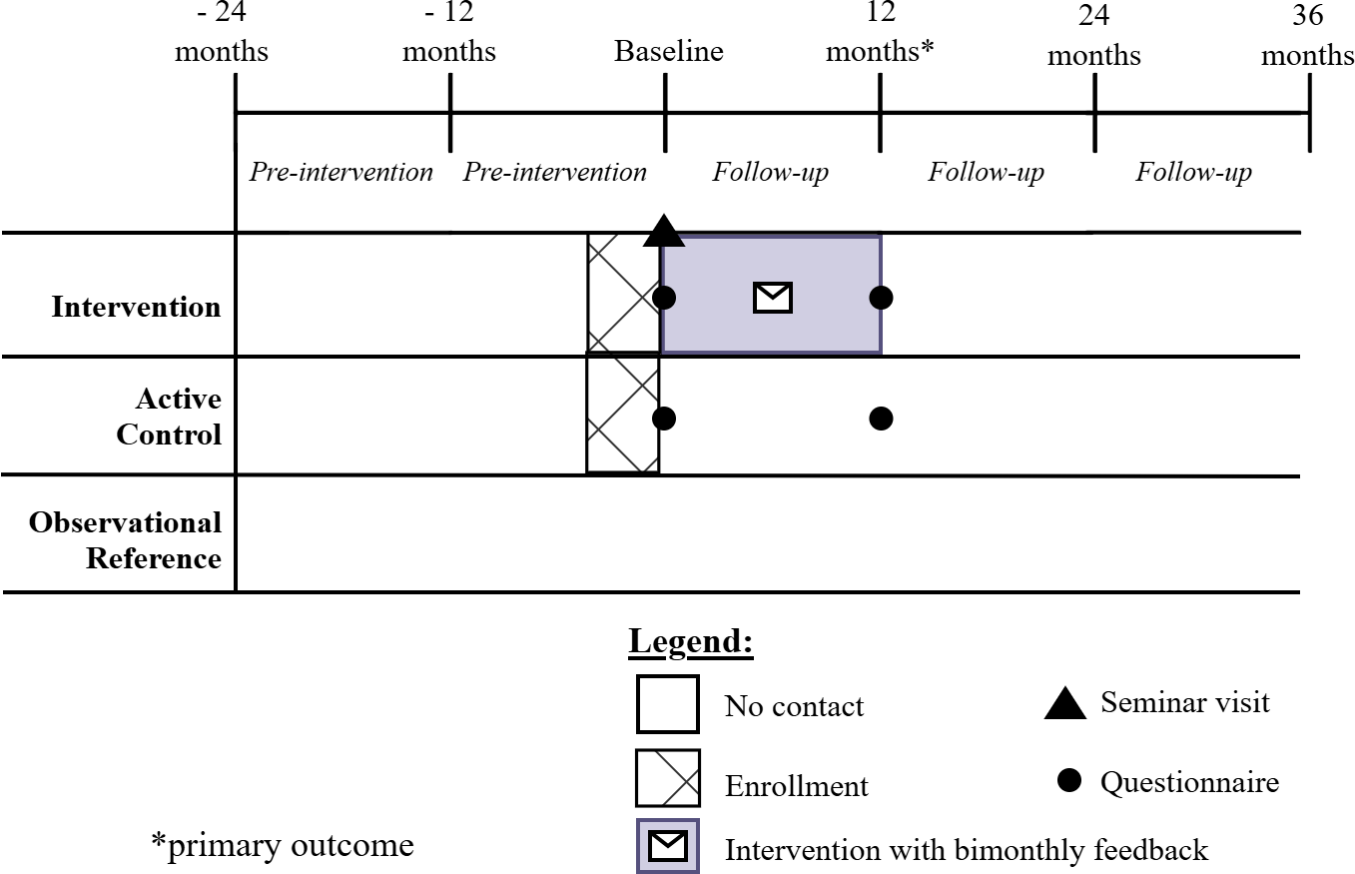
Schema of the *Smarta Val* pragmatic cluster randomised trial and study timepoints.

The duration of the intervention was 12 months from the date of the seminar visit, which was defined as the baseline date. Data on opioid prescriptions will be collected over a five-year period: two preintervention years prior to baseline and three years after baseline (figure 1). PHCC enrolment began in spring 2023, and seminar visits were conducted between June 2023 and February 2024. The final data extraction is planned for 36 months after the final seminar visit.

### Sample size

The outcome will be measured at the cluster level, using aggregated prescribing data for all registered patients at each PHCC. It was estimated that a minimum of 24 PHCCs per group were required to detect a 10% change in opioid prescribing between intervention and active control clinics. The calculation was based on a cluster-level regression analysis comparing change scores between groups, adjusted for baseline prescribing. Assumptions included 80% power, a two-sided significance level of 5%, and one covariate correlated with the outcome (r=0.39).

### Randomisation and blinding

#### Allocation to the intervention or the active control group

Consenting PHCCs will be enrolled on a rolling basis and randomised in a 1:1 parallel design to either the intervention or active control group. Randomisation will be pairwise, stratified by PHCC size, using computer-generated random numbers. PHCCs unmatched due to uneven numbers at a given time point will be carried forward to the next randomisation date, and any remaining unmatched PHCCs will be randomly allocated to either group.

#### Assignment of the GP delivering the intervention

At each randomisation time point, intervention PHCCs will also be randomised to one of two GPs who will conduct the seminar visit. Uneven allocation ratios (eg, 3:2) may be used due to differences in GP availability during the study period.

#### Assignment of baseline dates

PHCCs in the active control and observational reference groups will be assigned baseline dates to match intervention PHCC seminar visit dates. This ensures temporal alignment across groups and allows the leaflet to be sent to active control PHCCs on the same date as their corresponding intervention PHCCs.

#### Blinding

This is a pragmatic trial in which intervention assignment is known to both PHCCs and the study team. The statistician responsible for randomisation and outcome analyses will be blinded to PHCC names and the identity of the GP delivering the intervention. Analyses will be conducted using deidentified PHCC codes.

#### Data collection and management

Data for this study will be obtained from three sources: regional healthcare databases, questionnaires administered to PHCC managers and an intervention delivery form completed by study coordinators.

#### Regional healthcare databases

VAL is the name of the centralised data warehouse for monitoring healthcare utilisation in Region Stockholm.^37^ The regional healthcare databases in VAL include comprehensive healthcare records on all residents, including the PHCCs at which patients are registered, inpatient and outpatient visits, and prescriptions.

Data on the study population will be extracted from the databases for the five-year study period. At the level of the PHCC, variables include ownership type (public or private sector) and sociodemographic classification. Patient-level variables include data on PHCC registration, sociodemographic characteristics (eg, age, sex, Mosaic socioeconomic classification), and healthcare utilisation (eg, prescriptions, diagnoses and visit types).

Opioid prescribing will be quantified using dispensed DDDs, a WHO standard measure based on the assumed average maintenance dose for a drug’s main indication in adults,^38^ which is available in VAL. Key data to assess study outcomes include PHCC registration, prescribing PHCC, opioid ATC code, dispensation date, DDD per package, and the number of packages dispensed. Variables that will be used in the analysis of primary and secondary outcomes are listed in table 3. Additional variables such as migration events and date of death may also be included.

**Table 3:** Variables and descriptions from the regional healthcare databases (VAL) used to assess primary and secondary outcomes

Variable	Description
Registered patients per PHCC	Calculated from the number of individual registered patients at a PHCC each calendar month
Size of PHCC	Classification of PHCC size calculated from the number of registered patients: small (<5500), medium (5500–8500) or large (>8500)
PHCC registration	PHCC name, where a patient is registered each calendar month
ATC code	Seven-digit ATC code identifying the opioid substance prescribed
Prescribing PHCC	Name of the prescribing PHCC
Dispensation date	Date opioid was dispensed
Package DDD	DDDs per package dispensed
Number of packages	Number of packages dispensed

ATC, Anatomical Therapeutic Chemical; DDD, defined daily dose; PHCC, primary healthcare centre.

### Questionnaires

Questionnaires will be administered to PHCC managers in the intervention and active control groups at baseline (online supplemental table 1) and at 12-month follow-up (online supplemental tables 2,3) to assess contextual and intervention delivery-related factors.

Baseline questionnaires include the number and types of healthcare professionals (eg, GPs, nurses and psychologists/psychotherapists) employed by contract type, average number of registered patients per GP and rating of opioid prescribing routines at the PHCC. Follow-up questionnaires include the same question about rating of prescribing routines, but also ask about changes in prescribing routines, their experience of the intervention, use of study materials, personnel changes and involvement in non-intervention projects related to pain management or opioid prescription (eg, internships, specialist training and quality improvement projects).

### Intervention delivery form

The intervention delivery form will be filled out by one of two research coordinators at each seminar visit at baseline for intervention group PHCCs (online supplemental figure 3). It includes fields on attendance to assess reach, variables regarding the delivery of intervention, components to assess dose delivered and received, items to assess intervention fidelity and a section for observations.

### Data management

Data from questionnaires and forms will be entered into a secure electronic database. Deidentified data from the VAL regional healthcare databases will be delivered directly from the Centre for Health Data, Region Stockholm. All data will be stored and managed in accordance with Region Stockholm regulations and the EU General Data Protection Regulation. Only authorised study personnel will have access.

### Statistical methods

#### Descriptive analyses

Descriptive statistics will be used to summarise the characteristics of PHCCs and patient populations. Continuous variables will be presented as means with SDs, and categorical variables as frequencies with percentages. Baseline questionnaire data for the intervention and active control groups will also be summarised to highlight any differences between the groups. Open-ended responses will be analysed using qualitative methods.

#### Analyses of primary and secondary outcomes

Analyses of primary and secondary outcomes will be conducted at the PHCC level, with comparisons made between the intervention group and the active control group, as well as between the intervention group and the observational reference group. Patients registered at the PHCCs at any point during the 12 months before and 12 or 24 months after the intervention will be included in the analyses.

The number of dispensed DDDs of opioids and the number of patient-months (ie, the number of months during which patients are registered at a given PHCC) will be summarised for each PHCC. The change in DDDs per patient-month from baseline to follow-up will be calculated for each PHCC.

The difference in change between the groups will be estimated using linear regression, with baseline prescribing (DDDs per patient-month) and the matching variables accounted for in the analysis. Cluster effects at the patient level will not be adjusted for, as the unit of analysis is the PHCC. The assumption of normality will be assessed by inspecting the model residuals. Estimated effect sizes will be reported with p values (two-sided, α=0.05) and 95% CIs. A sensitivity analysis using morphine milligram equivalents may be used as a complement to DDDs to account for potency differences between opioid substances. Exploratory subgroup analyses based on characteristics of patients at participating PHCCs may also be conducted.

Cluster-level analyses will be based on register data from regional healthcare databases, which have full coverage of prescription data; missing data are not anticipated.

#### Analysis of intervention delivery

Intervention delivery will be examined by analysing data from the intervention delivery form (baseline) and questionnaires (baseline and 12-month follow-up). Descriptive summary statistics will be used to describe measures of reach, dose, intervention fidelity and acceptability. Observations and responses to open-ended questions will be analysed using qualitative methods.

### Patient and public involvement

No patient or public involvement is planned, although the intervention was developed by a multidisciplinary team of healthcare professionals and researchers.

### Ethics and dissemination

The study was approved by the Swedish Ethics Review Appeals Board on 20 April 2022 (Dnr 2021-06739-01) and was registered prospectively on ClinicalTrials.gov (NCT05577026). The study adheres to the principles of the Declaration of Helsinki. Participation in the study requires informed consent from PHCC managers in the intervention and active control groups. In accordance with national regulations, informed consent is not required from PHCCs in the observational reference group, as they are followed using register data only. No individual patients are recruited in the study as data on prescriptions are extracted solely from regional healthcare databases, which consist of anonymised data. Data from the VAL regional healthcare databases are not publicly available. Researchers may request access to the data by application to the Centre for Health Data, Region Stockholm. A data monitoring committee is not required due to minimal participant risk, use of database data and absence of investigational medicinal products. There are no discontinuation criteria in this pragmatic study.

Trial results will be reported in accordance with the Consolidated Standards of Reporting Trials (CONSORT) guidelines^39^ and will be published in international, peer-reviewed journals and presented at conferences.

## Discussion

This cluster randomised trial is the first to evaluate an education-based intervention to improve opioid prescribing practices in Swedish primary care. The intervention incorporates components that have been shown to be effective in prior research, including an educational seminar, written materials, and audit and feedback, and is designed to be time-efficient and scalable for routine care.

Use of regional healthcare databases enables longitudinal tracking of opioid prescribing over multiple years. The inclusion of intervention, active control and observational reference groups supports robust evaluation of intervention effects while accounting for secular trends. The extended follow-up and examination of intervention delivery may offer insights into long-term effects and implementation in routine practice.

The pragmatic design enables assessment of real-world opioid prescribing in primary care. However, this design involves less control over intervention fidelity, higher variability in implementation across PHCCs and potential contamination between PHCCs, which may affect internal validity. Additionally, the trial is conducted among high-prescribing PHCCs that opted to participate, which may limit generalisability. However, the inclusion of both an active control group and an observational reference group supports broader contextual interpretation and strengthens external validity.

Improving adherence to clinical guidelines in primary care has the potential to reduce inappropriate opioid prescription and associated harms. Given the central role of primary care in managing chronic pain and initiating opioid therapy, interventions targeting this clinical setting are particularly relevant. Findings from this study may contribute to the knowledge base on behavioural interventions and inform future strategies to support safe and evidence-based prescribing of opioids.

## Supplementary material

10.1136/bmjopen-2025-110818 online supplemental file 1
