# Supplementary material for: Improved opioid prescribing in primary care: protocol for a cluster randomised pragmatic trial
Source: BMJ Open. 2025 Dec 17;15(12):e110818. doi: 10.1136/bmjopen-2025-110818 (PMC12716515; doi:10.1136/bmjopen-2025-110818)
Supplement: online supplemental file 1 [file bmjopen-15-12-s001.docx]

# Supplemental Materials

| **Supplementary Table 1:** Baseline questionnaire for intervention and active control primary health care centers, answered by managers (translated to English) | |
| --- | --- |
| **Question** | **Response option** |
| 1. Name of the health center * | *Text with one line.* |
| 1. Name and title of the person who answered the questionnaire * | *Text with one line.* |
| 1. What year did the health center (HLM) start? * | *Text with one line.* |
| 1. How many listed patients does the health center have? * | *Text with one line.* |
| 1. How many physicians are there at the health center in total with permanent employment? *   *Enter number converted to full-time positions.* | *Text with one line.* |
| 1. How many physicians at the health center have temporary employment, including temporary physicians (hyrläkare), in total? * *Enter number converted to full-time positions.* | *Text with one line.* |
| 1. How many general practitioners with specialist training in General Practice (specialistläkare i allmänmedicin) (including residents/registrars; ST-läkare) are employed at the health center?   *Enter number converted to full-time positions.* | *Text with one line.* |
| 1. How many nurses are employed at the health center? *Enter number converted to full-time positions.* | *Text with one line.* |
| 1. How many psychologists or psychotherapists are employed at the health center? *Enter number converted to full-time positions.* | *Text with one line.* |
| 1. How many physiotherapists/physical therapists are employed at the health center? *Enter number converted to full-time positions.* | *Text with one line.* |
| 1. How do you feel that the routines for prescribing opioids worked at the primary health center during the last 12 months? *Choose the option that fits best.* | *Multiple choice, single answer:*  (5) Very good  (4) Good  (3) Fair  (2) Poor  (1) Very poor |
| 1. Other comment? | *Text with multiple lines.* |
| *required question | |

| **Supplementary Table 2:** Post-intervention/12-month questionnaire for intervention primary health care centers, answered by managers (translated to English) | |
| --- | --- |
| **Question** | **Response option** |
| 1. Name of the health center * | *Text with one line.* |
| 1. Name and title of the person who answered the questionnaire * | *Text with one line.* |
| 1. How do you feel that the routines for prescribing opioids worked at the health center during the last 12 months? *Choose the option that fits best.* | *Multiple choice, single answer:*  Very good  Good  Fair  Poor  Very poor |
| 1. Have you changed or updated your routines when prescribing opioids at the health center during the last 12 months? * | *Multiple choice, single answer:*  Yes  No, no change |
| 1. In what way have you changed or updated your routines for prescribing opioids at the health center?  *Please give some examples.* | *Text with multiple lines.* |
| 1. How did you experience of the intervention visit at your health center at the start of the project? * | *Multiple choice, single answer:*  (5) Very good  (4) Good  (3) Fair  (2) Bad  (1) Very bad |
| 1. At the beginning of the project, we sent you study materials. Which of these materials have you used at the health center in the past 12 months? * *You can choose one or more options.* | *Multiple choice, multiple answers:*  Patient information  Provider information  Powerpoint presentation  Examples of patient medical records  Have not used the materials  Other: *free text* |
| 1. In which ways have you used the materials at the health center? *   *You can choose one or more options.* | *Multiple choice, multiple answers:*  Was sent or handed out to health care professionals  Discussed at meetings  Shared with new health care professionals  Used in contact with the patient  Used as a basis for own materials  Have not used the material  Other: *free text* |
| 1. How has the feedback on opioid prescription that has been sent out every other month been used at the health center? * *You can choose one or more options.* | *Multiple choice, multiple answers:*  Was sent or handed out to health care professionals  Discussed at meetings  Shared with new health care professionals  Used in contact with the patient  Used as a basis for own materials  Have not used the material  Other: *free text* |
| 1. Has the health center received any continuing education or training about pain/opioids in the last 12 months (besides this study)? * | *Multiple choice, single answer:*  Yes  No |
| 1. Has the health center undertaken a quality improvement project (kvalitetsarbete) or other improvement project on pain/opioids in the last 12 months? * | *Multiple choice, single answer:*  Yes  No |
| 1. If yes, please name the type of project | *Text with multiple lines.* |
| 1. How has staffing of health care professionals been at the health center during the past 12 months? * | *Multiple choice, single answer:*  Fully staffed  Understaffed  Other: *free text* |
| 1. Other comments or questions? | *Text with multiple lines.* |
| *required question | |

| **Supplementary Table 3:** Post-intervention/12-month questionnaire for active control primary health care centers, answered by managers (translated to English) | |
| --- | --- |
| **Question** | **Response option** |
| 1. Name of the health center * | *Text with one line.* |
| 1. Name and title of the person who answered the questionnaire * | *Text with one line.* |
| 1. How do you feel that the routines for prescribing opioids worked at the health center during the last 12 months? *Choose the option that fits best.* | *Multiple choice, single answer:*  Very good  Good  Fair  Poor  Very poor |
| 1. Have you changed or updated your routines when prescribing opioids at the health center during the last 12 months? * | *Multiple choice, single answer:*  Yes  No, no change |
| 1. In what way have you changed or updated your routines for prescribing opioids at the health center?  *Please give some examples.* | *Text with multiple lines.* |
| 1. At the beginning of the project, we sent you a study material. In what way have you used the material at the health center in the last 12 months? * *You can choose one or more options.* | *Multiple choice, multiple answers:*  Was sent/handed out to health care professionals  Discussed at meetings  Shared with new health care professionals  Used as a basis for own material  Used in contact with the patient  Have not used the material  Other: *free text* |
| 1. Has the health center received any continuing education or training about pain/opioids in the last 12 months (besides this study)? * | *Multiple choice, single answer:*  Yes  No |
| 1. Has the health center undertaken a quality improvement project (kvalitetsarbete) or other improvement project on pain/opioids in the last 12 months? * | *Multiple choice, single answer:*  Yes  No |
| 1. If yes, please name the type of project | *Text with multiple lines.* |
| 1. How has staffing of medical personnel been at the health center during the past 12 months? * | *Multiple choice, single answer:*  Fully staffed  Understaffed  Other: *free text* |
| 1. Other comments or questions? | *Text with multiple lines.* |
| *required question | |

**Supplementary Figure 1:** Informed consent form from the Smarta Val trial (Original, in Swedish)

**Information till verksamhetschefer**

Inbjudan till deltagande i projekt om klok förskrivning av opioider i primärvården: en randomiserad kontrollerad studie

**Vi vill fråga dig i egenskap av verksamhetschef om er vårdcentral vill deltaga i ett forskningsprojekt?**

I det här dokumentet får du information om projektet och om vad det innebär att delta. Vid eventuella frågor eller funderingar är du välkommen att kontakta ansvariga för projektet.

**Vad är det för projekt och varför vill ni att vår vårdcentral ska delta?**

I vår kliniska vardag står vi ofta inför medicinska och etiska dilemman vid förskrivning av narkotiska läkemedel så som opioider. Målet med projektet är en uppnå en klok och säker behandling med opioider för smärta på vårdcentralen.

**Studiens syfte**

Syftet är att i en randomiserad kontrollerad studie utveckla och utvärdera om en riktad utbildningsintervention samt återkoppling av förskrivningsdata kan öka kvaliteten på behandling med opioider för smärta, mätt som skillnad i förskrivning, på vårdcentraler i interventionsgruppen jämfört med vårdcentraler i kontrollgruppen.

**Målgrupp**

Interventionen riktar sig till vårdcentralen inklusive verksamhetschefer, läkare och annan vårdpersonal på vårdcentralen.

**Vilka vårdcentraler kan delta?**

För att delta i studien krävs att er vårdcentral har:

- Minst 3000 listade patienter
- Minst två fast anställda läkare som arbetar minst 75% vardera
- Vårdavtal med regionen
- Journalsystem med anslutning till regionens centrala datalager
- Att vårdcentralen har funnits i minst 12 månader (dvs. inte är nyöppnad)

**Hur väljs interventionsgrupp och kontrollgrupp?**

Om er vårdcentral kommer tillhöra interventionsgrupp eller kontrollgrupp avgörs genom lottning där hälften av vårdcentralerna lottas till interventionsgrupp och den andra hälften till kontrollgrupp.

Version 1.0, 2022-03-30 1

**Vad får interventionsgruppen?**

Utbildningsinterventionen omfattar ett utbildningstillfälle (cirka 90 min) för vårdpersonal vid enheten samt regelbunden återkoppling av vårdcentralens förskrivningsdata under 12 månader. Interventionen bygger på riktlinjer, evidens och beprövade erfarenheter gällande förskrivning av opioider vid smärta inom primärvården. Vårdcentralen kommer att få information samt stöd i arbetet kring rutiner på vårdcentralen.

**Vad får kontrollgruppen?**

Verksamhetscheferna på dessa vårdcentraler kommer få skriftlig information om aktuella riktlinjer för behandling med opioider. Interventionen bygger på riktlinjer, evidens och beprövade erfarenheter gällande förskrivning av opioider inom primärvården.

**Hur går datainsamlingen till?**

Inga patienter kommer att kontaktas för datainsamling. För att utvärdera resultat inhämtas registerliknande information från regionens centrala hälsodatalager, VAL-databasen och Nationella läkemedelsregistret och andra hälsodataregister på Socialstyrelsen. Information på patientnivå inhämtas om läkemedel (eg. preparatnamn, ATC-kod, dos, beredning, datum) samt ålder, kön, diagnoser, listning/vårdcentral, vårdkonsumtion, åtgärd (via KVÅ-koder), mortalitet, socioekonomisk status och region. Data om vårdcentralen kommer samlas in manuellt om driftform, listning, täckningsgrad, och bemanning. Datainsamling för både interventionsgrupp och kontrollgrupp sker under 48 månader.

**Vem är forskningshuvudman?**

Forskningshuvudman för studien är Stockholms läns sjukvårdsområde (SLSO), Region Stockholm. Med forskningshuvudman menas den myndighet som är ansvarig för studien.

**Hantering av data och sekretess**

SLSO är en myndighet som har skyldighet att bl.a. följa reglerna för allmänna handlingar, myndigheters arkiv och offentlig statistik. Studien använder avidentifierade information från regionens centrala datalager och nationella register som skyddas av bestämmelser om sekretess enligt offentlighets- och sekretesslagen. Registerdata kommer vara avidentifierade för forskarna redan vid datautlämningen så att inga enskilda individer går att identifiera. Inom VAL-databaserna krypteras personnummer för varje patient till ett unikt ID-nummer. Dekryptering av personnumren är inte möjlig. Studiematerial kommer att förstöras efter en viss tids arkivering i enlighet med gällande lagstiftning för myndigheten. Om oväntade allvarliga bifynd uppkommer i studien så kommer ansvarig forskare kontakta verksamhetschef på respektive vårdcentral.

**Möjliga följder och risker med deltagande**

Det finns en viss integritetsrisk i att studera förskrivning baserat på befintlig registerinformation som inhämtas rutinmässigt från journalsystem. Samtliga analyser kommer dock att göras med data avidentifierade data grupperat på en sådan nivå att ingen information kan härledas till någon specifik individ. Liknande analyser av förskrivningsdata görs sedan flera år i regionen som del av vårdens kvalitetsuppföljning. Enskilda läkares förskrivning kommer inte kunna röjas och resultaten i studien kommer i huvudsak presenteras på vårdcentralsnivå.

Det finns en potentiell risk med överförskrivning av opioider men en allt för restriktiv förskrivning skulle också kunna leda till risker. Opioider har en legitim och viktig roll i vården om de avvänds på rätt sätt i linje med rådande riktlinjer och rekommendationer. Projektet förespråkar således inte minskad förskrivning överlag utan en klok och säker förskrivning av opioider i relation till riktlinjer och behandlingsrekommendationer.

Version 1.0, 2022-03-30 2

**Möjlig nytta**

Om interventionen är effektiv kan den bidra till att uppnå en klok och säker behandling med opioider och därmed ge direkt nytta för vårdcentralen.

**Rätten att inge klagomål gällande behandlingen av personuppgifter**

Medverkande enheter har rätt att kontakta dataskyddsombud för Stockholms läns sjukvårdsområde på gdpr.slso@sll.se eller via växelnummer 08-123 400 00 för frågor som rör behandling av personuppgifter i studien. Vid synpunkter på hur personuppgifter behandlas finns rätt att ge in klagomål till Datainspektionen, som är tillsynsmyndighet.

**Information om resultatet av studien**

Under studiens gång finns möjlighet att få löpande information genom att kontakta ansvarig forskare. Resultat från studien planeras att publiceras vetenskapligt samt spridas inom nätverk och på konferenser.

**Försäkring och ersättning**

Ingen ersättning utgår i studien.

**Deltagandet är frivilligt**

Deltagande i studien är frivilligt. Ni kan när som helst välja att avbryta deltagandet utan att uppge några skäl. Vid avbrytande av deltagande, kontakta ansvariga för studien.

**Ansvarig för studien**

Johan Franck, Professor

Beroendecentrum Stockholm

Friskvårdsvägen 6

113 65 Stockholm

[johan.franck@regionstockholm.se](mailto:johan.franck@regionstockholm.se)

**Projektledare**

Jeanette Westman, Professor

Akademiskt Primärvårdscenter

Solnavägen 1E

113 65 Stockholm

[jeanette.westman@regionstockholm.se](mailto:jeanette.westman@regionstockholm.se)

**Forskningskoordinator**

Cecilia Krüger

Beroendecentrum Stockholm

Tantogatan 8

118 67, Stockholm

[cecilia.kruger@regionstockholm.se](mailto:cecilia.kruger@regionstockholm.se)

070-180-1778

Version 1.0, 2022-03-30 3

**Samtycke till att delta i studien (verksamhetschef)**

Jag har fått muntlig och skriftlig information om studien och har haft möjlighet att ställa frågor. Jag får behålla den skriftliga informationen.

☐ I egenskap av chef för verksamheten samtycker jag till att vårdcentralen/enheten deltar i projektet om klok förskrivning av opioider som presenterats ovan.

Ort och datum:_____________________________________________________

Verksamhet:__________________________________________________________

Namnteckning: _____________________________________________________

Namnförtydligande:__________________________________________________

Personnummer: -

Version 1.0, 2022-03-30 4

**Supplementary Figure 2:** Example of email follow-up containing audit-and-feedback materials in the Smarta Val trial, delivered to experimental group primary health care centers bi-monthly over 12 months (translated to English).

**SUBJECT:** Follow-up on opioid prescribing at your healthcare center – Smarta Val

Hello,

Thank you for your health care center’s participation in the “Smarta Val” project, which focuses on safe and appropriate opioid prescribing in primary care. It is now time for your next follow-up in the study!

Below are two figures showing the prescribing patterns of opioids at your primary healthcare center since the educational seminar visit (indicated by the dashed line). Opioids are measured in Defined Daily Doses (DDD). One DDD corresponds to 100 mg of morphine.

**Figure 1:**  Total amount of prescribed opioids (N02A) in DDD


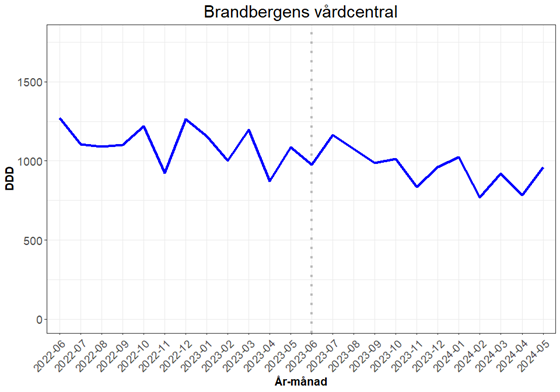


**Figure 2:**  Total amount of prescribed opioids broken down by specific substances (ATC code)


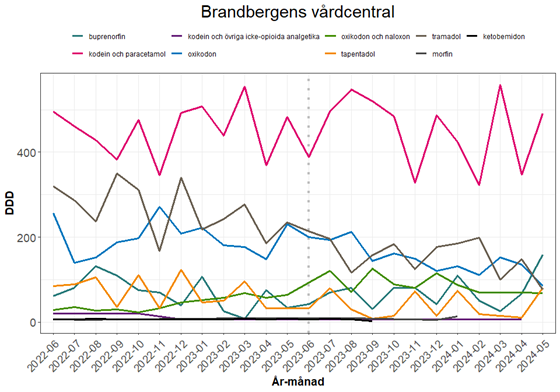


We will continue to send out prescribing data every other month, so keep an eye out for the next email!

*Best regards,*

The Smarta Val research team
[smarta.val.slso@regionstockholm.se](mailto:smarta.val.slso@regionstockholm.se)


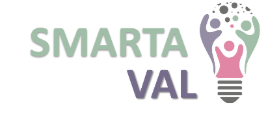


**Supplementary Figure 3:** Intervention delivery form, scored by one of two research coordinators at the baseline during the seminar visit to intervention group PHCCs (original, in English)

**Intervention Delivery Form, Smarta Val**
Intervention Group

Location: _________________________________ Date: __________________________

Time start: ________________________________ Time end: _________________________

| **Attendance** | **Yes** | **No** |
| --- | --- | --- |
| Distribution of attendance record |  |  |
| Number of attending staff and roll |  |  |
| Doctors | *nr.* | |
| Nurses | *nr.* | |
| Other professions *(record which)* | *nr.* | |
|  | *Tot nr.* | |
| **Delivery of short survey to manager** | **Yes** | **No** |
| Gave survey (paper copy) |  |  |
| **Presentation** | **Yes** | **No** |
| Follow PowerPoint presentation and slides (academic detailing) |  |  |
| - Evidence from the literature |  |  |
| Show film |  |  |
| Show information on tapering / referral |  |  |
| Show benchmarking prescription data |  |  |
| - *Est. total nr minutes discussed* |  | |
| Discussion of strategies at the clinic to reduce inappropriate prescription |  |  |
| - *Est. total nr minutes discussed* |  | |
| **Circulation of patient / provider materials** | **Yes** | **No** |
| - Written information for the provider |  |  |
| - Written information for the patient (short and long versions) |  |  |
| - Example treatment and follow-up plans |  |  |
| Reminder to not share documents across clinics |  |  |
| Total (out of 13) |  | |

Observations/field notes:

| **Ongoing external education in the field?** *(to be completed post-intervention)* | **Yes** | **No** |
| --- | --- | --- |
| Pain education workshop through the Pain Team? |  |  |
| Quality improvement project within the field? *If yes, topic:* |  |  |
| Other? *(if yes, describe)* |  |  |
