# Supplementary material for: Improved opioid prescribing in primary care: protocol for a cluster randomised pragmatic trial
Source: BMJ Open. 2025 Dec 17;15(12):e110818. doi: 10.1136/bmjopen-2025-110818 (PMC12716515; doi:10.1136/bmjopen-2025-110818)
Supplement: Supplementary file 2 [file bmjopen-2025-110818.reviewer_comments.pdf]

PEER REVIEW HISTORY

BMJ Open publishes all reviews undertaken for accepted manuscripts. Reviewers are asked to complete a checklist review form and are provided with free text boxes to elaborate on their assessment. These free text comments are reproduced below.

ARTICLE DETAILS

Title (Provisional)

Improved opioid prescribing in primary care: protocol for a cluster randomized pragmatic trial

Authors

Krüger, Cecilia; Franck, J; Hällgren, Jonas; af Winklerfelt Hammarberg, Sandra; Norman, Christer; Niper, Åsa; Westman, Jeanette

---

VERSION 1 - REVIEW

**Reviewer**	**1**
**Name**	**Abdel Shaheed, Christina**
**Affiliation**	**University of Sydney Sydney Medical School, Public Health**
**Date**	**02-Nov-2025**
**COI**	**None**

Thank you for the opportunity to review the manuscript entitled: Improved Opioid Prescribing in Primary Care: Protocol for a Cluster Randomized Pragmatic Trial

The introduction sets the scene for the study and the need for it.

I have some suggestions for the methods section for the authors to consider.

Please specify the target patient population. Is this people with acute and chronic pain who are prescribed an opioid? What is the eligibility criteria for patient inclusion? Will any patient reported outcomes be collected? Or is it only at the practice and GP level?

Harms is a mandatory outcome that needs to be reported in clinical trials (refer to CONSORT 2025). Can the authors please described how harms data will be collected, and please add this to the protocol?

I felt a little more description on the inferential statistics planned is necessary.

Is the 90 minute educational seminar for the GPs? This may just need to be more clearly described in the methods.

Will cluster randomisation account for geographical location (e.g urban/ rural)?

What written materials will be provided to patients in the intervention and control arms? Are these the same or will they differ?

Under sample size calculations, how many patient participants need to be enrolled?

Under primary outcome, what type of change are you looking for? A reduction in DDD? May be worth making this clearer.

For secondary outcomes, it would also be worth making these clearer. What happens if the change to another opioid substance results in higher cumulative opioid dose (in morphine milligram equivalents).

VERSION 1 - AUTHOR RESPONSE

Reviewer: 1
Dr. Christina Abdel Shaheed, University of Sydney, Sydney Medical School
Comments to the Author:

Thank you for the opportunity to review the manuscript entitled: Improved Opioid Prescribing in Primary Care: Protocol for a Cluster Randomized Pragmatic Trial.

Thank you for agreeing to review our manuscript. We appreciate your time and effort in providing comments, which we feel have helped improve the quality and clarity of the protocol.

The introduction sets the scene for the study and the need for it.

Thank you.

I have some suggestions for the methods section for the authors to consider. Please specify the target patient population. Is this people with acute and chronic pain who are prescribed an opioid? What is the eligibility criteria for patient

inclusion? Will any patient reported outcomes be collected? Or is it only at the practice and GP level?

Thank you for these helpful suggestions.

The intervention is recruited, randomized, and implemented at the cluster level. The intervention targets all PHCC health care professionals, with a particular focus on prescribers. The text describing this in the Methods under “Intervention group” has been revised for clarity (page 6, lines 149–153).

No patients are directly recruited into the trial. No patient-reported outcomes will be collected. However, anonymized data on individual-level opioid prescriptions for all adult registered patients at participating PHCCs will be used to assess outcomes at the cluster (PHCC) level. Indication for opioid prescription (e.g., acute, chronic pain) was not used in population selection. The text under “Design” in the Methods section has been revised to more clearly describe the methods for the reader early on (page 5, lines 113–116).

Harms is a mandatory outcome that needs to be reported in clinical trials (refer to CONSORT 2025). Can the authors please described how harms data will be collected, and please add this to the protocol?

Thank you for highlighting the need to describe harm assessment more clearly. In response, we have moved the original text on harms from the Discussion section into a dedicated subheading, “Harms”, following “Outcomes” in the Methods section (page 8, lines 205–213), in accordance with SPIRIT guidelines for trial protocols.

We clarified that standard routines for monitoring patient safety are in place at each PHCC, and PHCC personnel also have the option to report any harms directly to the study team (page 8, lines 206–209). As the intervention consists solely of an educational seminar, prescribing recommendations, and feedback at the PHCC level, risks to health care professionals and patients are anticipated to be minimal, and no systematic harm assessment will be conducted.

Any harms reported during the trial will be documented and presented in future outcome publications, in alignment with CONSORT requirements.

I felt a little more description on the inferential statistics planned is necessary.

Thank you for this suggestion. We have expanded the “Statistical Methods” section to provide additional detail on the planned inferential analyses, “Analysis of primary and secondary outcomes” (page 10, lines 298-315). Specifically, we now clarify that:

- Analyses of primary and secondary outcomes will be conducted at the PHCC level using linear regression, adjusting for baseline prescribing and matching variables.
- Cluster effects at the patient level will not be adjusted for as the unit of analysis is the PHCC (line 308).
- Estimated effect sizes will be reported with p values (two-sided, $\alpha = 0.05$) and 95% confidence intervals (line 310).

- Missing data are not expected for the primary outcome as register-based data are used (lines 314-315).
- A sensitivity analysis using morphine milligram equivalents (MMEs) may be used (lines 310-312).
- Subgroup analyses will be exploratory (line 312).

These additions aim to provide greater transparency on the inferential approach while maintaining clarity and alignment with SPIRIT reporting guidelines.

Is the 90 minute educational seminar for the GPs? This may just need to be more clearly described in the methods.

Thank you for this helpful comment. We have clarified the description of the intervention in the Methods section to make it explicit that the 90-minute educational seminar is primarily intended for general practitioners, although other health care professionals with patient contact are also encouraged to attend (page 6, lines 150-153).

Will cluster randomisation account for geographical location (e.g urban/ rural)?

Thank you for this important question. We agree that accounting for urban/rural location or other sociodemographic factors during randomization could have been of interest. However, given the relatively small sample size, it was not feasible to include multiple stratification variables. Therefore, PHCC size was selected as the sole stratification variable.

We would like to note that the study collects data on sociodemographic characteristics of PHCCs and registered patients (see page 9, lines 257–261) which will be reported in future analyses.

What written materials will be provided to patients in the intervention and control arms? Are these the same or will they differ?

Thank you for highlighting the need to clarify differences in which materials are provided to the intervention and active control groups, and who the target is. All study materials were distributed to health care personnel at participating PHCCs – no materials were provided directly to patients. We have made minor revisions throughout the Methods to make this explicit (pages 5-9).

The intervention group receives several types of materials, including patient record templates and a summary of Region Stockholm's Wise List prescribing recommendations for use by providers, and patient–provider agreements which are given to providers but may potentially be used with patients. Revisions have been incorporated under the "Intervention group" header to make the intervention group study materials and their sources clearer (page 6, lines 149-150 and 156-58; page 7, lines 167-168, 171–177).

The active control group receives only a single-page leaflet summarizing Wise List recommendations and providing references and links to national and regional treatment guidelines (Swedish Medical Products Agency and Viss). This is now also clarified in revisions (page 7, lines 183–186).

Corresponding edits have also been made in Table 2 (page 6). Additionally, the terms used to describe the materials for each group (i.e., “leaflet” for the active control and “written materials” for the intervention group) have been harmonized throughout the manuscript, resulting in minor edits (i.e., lines 26, 32, 52, 108, 149, 242).

Under sample size calculations, how many patient participants need to be enrolled?

Thank you for your question. The sample size calculation was performed at the cluster level because both randomization and the primary outcome are analyzed at the PHCC level, not at the individual patient level. Each PHCC has at least 3,000 registered patients, and the analysis will use aggregated prescribing data for all registered patients at each PHCC. We have added a clarifying statement to the start of the “Sample size” section (page 8, lines 222-223) to make this explicit.

Under primary outcome, what type of change are you looking for? A reduction in DDD? May be worth making this clearer.

Thank you for this comment. The outcomes were intentionally worded neutrally to capture any change in opioid prescribing, whether an increase or a decrease, over time. This wording, using “change” instead of “reduction”, was also preferred by the Swedish Ethical Review Authority to allow for both possibilities.

For secondary outcomes, it would also be worth making these clearer. What happens if the change to another opioid substance results in higher cumulative opioid dose (in morphine milligram equivalents).

Thank you for this thoughtful suggestion.

We selected defined daily dose (DDD) as the primary outcome because it provides a standardized measure that normalizes consumption across different opioid substances and is readily available for each dispensation in the VAL databases. To clarify this for readers, we have added a sentence defining DDD in the “Data collection and management” section (page 9, lines 262-264).

We acknowledge your concern that DDD does not account for potency differences between different substances. It is possible that similar DDDs could mask changes in overall opioid exposure if patients are switched to opioids with different potency over time. We recognize the value of presenting results in morphine milligram equivalents (MMEs) to address this risk, although this approach requires assumptions about conversion factors. We have therefore added a statement in the statistical methods section indicating that MME may be included as an additional metric in sensitivity analyses future publications (page 11, lines 310-312).

*** ***

COI statement(s):

Reviewer: 1
If you have selected ‘Yes’ above, please provide details of any competing interests.:
NA.
